# The chemical, mechanical, and physical properties of 3D printed materials composed of TiO_2_-ABS nanocomposites

**DOI:** 10.1080/14686996.2016.1152879

**Published:** 2016-04-01

**Authors:** Matthew R. Skorski, Jake M. Esenther, Zeeshan Ahmed, Abigail E. Miller, Matthew R. Hartings

**Affiliations:** ^a^ Department of Chemistry, American University, 4400 Massachusetts Ave., NW, Washington, DC, 20016, USA; ^b^ Thermodynamic Metrology Group, Sensor Science Division, Physical Measurement Laboratory, National Institute of Standards and Technology, Gaithersburg, MD20899, USA; ^c^ Food and Drug Administration, Washington, DC, USA

**Keywords:** 3D printing, nanocomposite, TiO_2_ nanoparticle, ABS, photocatalysis, 20 Organic and soft materials (colloids, liquid crystals, gel, polymers)

## Abstract

To expand the chemical capabilities of 3D printed structures generated from commercial thermoplastic printers, we have produced and printed polymer filaments that contain inorganic nanoparticles. TiO_2_ was dispersed into acrylonitrile butadiene styrene (ABS) and extruded into filaments with 1.75 mm diameters. We produced filaments with TiO_2_ compositions of 1, 5, and 10% (kg/kg) and printed structures using a commercial 3D printer. Our experiments suggest that ABS undergoes minor degradation in the presence of TiO_2_ during the different processing steps. The measured mechanical properties (strain and Young’s modulus) for all of the composites are similar to those of structures printed from the pure polymer. TiO_2_ incorporation at 1% negatively affects the stress at breaking point and the flexural stress. Structures produced from the 5 and 10% nanocomposites display a higher breaking point stress than those printed from the pure polymer. TiO_2_ within the printed matrix was able to quench the intrinsic fluorescence of the polymer. TiO_2_ was also able to photocatalyze the degradation of a rhodamine 6G in solution. These experiments display chemical reactivity in nanocomposites that are printed using commercial 3D printers, and we expect that our methodology will help to inform others who seek to incorporate catalytic nanoparticles in 3D printed structures.

## Graphical abstract

**Figure:**
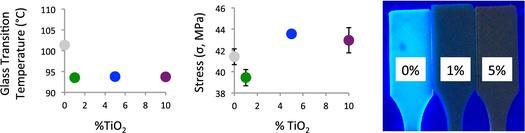

## 1. Introduction

Additive manufacturing, or three-dimensional printing, encompasses a number of techniques in which a material is deposited in a layer-by-layer fashion to produce a larger 3D structure.[1, 2] A diversity of materials (sugars,[3] thermoplastics, photopolymers,[4] glass,[5] metals,[6] metal oxides,[7] and ceramics,[8] to name a few) are used to produce 3D printed figures. Of these, thermoplastics are the most common for home enthusiasts and experimental laboratories due to the large number of commercially available printers that employ these materials, which results from the safety, ease of use, and cost of the equipment. Thermoplastics are extruded at temperatures above their glass transition temperature (T_g_), at which they display increased flexibility and flow properties.

Commercial thermoplastic 3D printers have found their way into several research laboratories. Despite their popularity, their use has been mostly confined to producing tools that assist in performing research rather than producing structures that are subjects of research. This is especially true for the chemical sciences. Taken from a chemist’s point of view, a printed item is primarily an inert object.

That is not to say that chemists and (bio)materials scientists have not found innovative ways to use 3D printing to advance their fields. Cronin and co-workers have developed 3D printed reactionware using acetoxy silicone polymers that are normally used as household sealants.[9–11] Their reactionware has been used to facilitate syntheses, purifications, crystallizations, and analyses. Another subject where 3D printing has great potential is in the area of tissue engineering. Several groups of scientists have printed hydrogels that have been seeded with mammalian cells.[12–14] The hydrogels are fixed into place with UV light, and the cells are allowed to culture within the printed scaffold. In each of these examples, a polymer with optimal flow properties at room temperature is extruded and induced to set through some external stimulus (light, oxygen, etc.).

While 3D printing holds great promise for advancing chemistry and materials science, this promise has not been realized for the ubiquitous thermoplastic printers. We contend that one way to imbue active chemistry into 3D printed thermoplastic objects is to incorporate inorganic nanoparticles into the polymer filaments. Because organic dye molecules are infused into a polymeric matrix to create different colored filaments, inorganic nanoparticles should be able to be integrated in much the same way. The functionalities associated with different types of nanoparticles (catalysis,[15] luminescence,[15] thermal [16] and electric [17] conductivity, and gas storage,[18] to name a few) should retain their properties when incorporated into a thermoplastic. We have incorporated TiO_2_ nanoparticles into acrylonitrile butadiene styrene (ABS) filaments. The TiO_2_-ABS filaments were printed in multiple shapes and tested for their mechanical, physical, and chemical properties. TiO_2_-polymer nanocomposites have been studied in detail.[19–24] Nanocomposites are materials in which nanoscale particles have been incorporated into a polymer matrix. In many nanocomposites, advantageous properties of the nanoparticles and the polymer are maintained in the bulk material. In some nanocomposites, enhancement of functional properties is observed. TiO_2_ has been shown to increase the mechanical strength of the nanocomposite over the pure polymer.[20, 22, 24] TiO_2_ can increase the dielectric properties of a polymer for use in insulating materials.[21] TiO_2_ nanocomposites can also photocatalytically degrade environmental organic pollutants.[24] 3D printed TiO_2_-ABS nanocomposites should follow similar trends, and 3D printing of this material could become a viable way to fabricate custom, on-demand parts that can meet any number of these technological needs. In the experiments presented here, we aim to determine if printed TiO_2_-ABS composites maintain the advantageous properties of the nanocomposites as described above.

TiO_2_ nanoparticles make an ideal test case for an inorganic nanoparticle filler within thermoplastic printing filaments. TiO_2_ is a known photocatalyst, capable of generating free radical species in both aqueous and organic solvents and in the presence of oxygen upon irradiation.[25] The band gap of TiO_2_ corresponds to UV wavelengths. Upon absorbance of light, electrons in the conduction band can form superoxide radicals (O^2-^•) from adsorbed oxygen, and holes in the valence band can form hydroxyl radicals (•OH) from water. Because of these properties, TiO_2_ has potential applications in the photocatalytic removal of pollution from air, water, and agricultural sources.[26, 27] It also has potential uses in solar energy [28] and solar hydrogen production.[29] The use of TiO_2_ nanoparticles in many consumer products, such as paints, means that this material is abundant and can be acquired at low cost. All of these factors – low cost, ease of purchase, and readily testable chemical properties – make TiO_2_ an ideal test case for producing functional nanocomposite ABS filaments.

We present here detailed observations of several chemical, physical, and mechanical properties of 3D printed TiO_2_-ABS nanocomposites. We describe the preparation and evaluation of a range of TiO_2_-ABS compositions (0, 1, 5, and 10% kg/kg). There is one previous study of the mechanical properties of printed 5% TiO_2_-ABS structures.[22] Our measurements generally agree with the previous observations and expand upon the analysis of how TiO_2_ affects the properties of printed ABS. As there are few studies of 3D printed TiO_2_-polymer nanocomposites, we have attempted to understand how the different processing steps affect the chemical and physical properties of the polymer. We determined the effect of TiO_2_ on the thermal transitions and the molecular weight of the processed (solvent cast, extruded, and printed) ABS. We measured the ability of the TiO_2_ to quench the luminescence that arises from the styrene functional groups within ABS. Finally, we analyzed the photocatalytic degradation of rhodamine 6G by 3D printed structures of TiO_2_-ABS nanocomposites. We expect that these experiments will inform future studies in which nanoparticles are incorporated into thermoplastic 3D printing filaments for developing chemically active 3D printed materials.

## 2. Materials and methods

### 2.1. Materials

Titanium(IV) oxide (nanopowder, 21 nm particle size by transmission electron microscopy, 80–90% anatase polyform with small percentage of rutile polyform) and rhodamine 6G were purchased from Sigma Aldrich (St Louis, Missouri, USA).[30] Commercial ABS filament was purchased from Octave Systems (Santa Clara, California, USA) (natural color, 1.75 mm width). High Performance Liquid Chromatography (HPLC)-grade water, dimethylformamide (DMF), and acetone were purchased from BDH Chemicals (Radnor, Pennsylvania, USA). ABS pellets (resin: GPA 100; color #: NC010; color: natural; lot #: C14–0702 K) was acquired from LTL Color Compounders, Inc. LiBr was purchased from Acros Chemicals (New Jersey, USA).

### 2.2. Production and printing of TiO_2_-ABS filaments

ABS and TiO_2_ mixtures (0, 1, 5, and 10% TiO_2_ by dry weight) were suspended and dispersed in acetone (in a sealed container) over several hours at 35°C in a sonicator (VWR Symphony ultrasonic cleaner, VWR, Inc., Radnor, Pennsylvania, USA). The highest percentage of TiO_2_ used in this study was 10% (kg/kg) because we found that nanocomposites produced at higher percentages were incompatible with our printing process. A typical batch included 20 total grams of ABS and TiO_2_ in 200 ml of acetone. As an example, a 1% sample was made by suspending 0.2 g of TiO_2_ and 19.8 g of ABS in 200 ml of acetone, held in an Erlenmeyer flask, and agitated in a heated sonicating bath. The dispersed samples were solvent cast in Teflon coated, aluminum frying pans (used for their large surface area). The pans were placed on hot plates set to 80 °C, and the solvent was evaporated. Solvent casting by heating samples in large surface area pans is necessary to reduce the amount of TiO_2_ clumping observed in slower evaporation processes.

The films were cut into small squares (roughly 1 cm by 1 cm) and extruded with a DSM Xplore Micro 15 cc Twin Screw Compounder (DSM Xplore, Geleen, the Netherlands). The filaments produced using this compounder were too wide to be compatible with the 3D printer. These filaments were cut into small pieces using a wire cutter and extruded into a 1.75 mm-wide filament using a Filabot Wee Extruder from Filabot (Barre, Vermont, USA). These filaments were used to print various structures by a Flashforge Creator 3D Printer from Flashforge (Rowland Heights, California, USA) with dual extruders.

Two different shapes (dogbones and cylinders) were printed using the factory settings for ABS filaments. The dogbone shape and dimensions were printed to match the specification described by the American Society for Testing and Materials (ASTM) standard D638.[31] A diagram of these dimensions is shown in the Supporting Information. The cylinders were 2.5 cm in diameter, designed to fit in the solid sample holder used in fluorescence measurements. Both shapes were printed at a 10% fill volume and without a raft. The absence of a raft led to a smoother surface, which facilitated different spectroscopic measurements.

### 2.3. Presence of TiO_2_ in ABS samples

Powder X-ray diffraction (XRD) measurements were used to confirm the presence of TiO_2_ within the polymer nanocomposite. XRD measurements were recorded using a Rigaku Miniflex II equipped with an NaI scintillation counter detector, a 450 W Cu K α (*λ* = 0.1540562 nm) X-ray source, and a diffracted beam monochromator. Each sample was mounted on an aluminum holder.

### 2.4. Effect of TiO_2_ and processing on ABS structure

To determine if processing (solvent casting, extruding, and printing) ABS in the presence of TiO_2_ affects the polymer structure, we measured monomer content with Fourier transform infrared spectroscopy (FTIR), ABS thermal transitions with differential scanning calorimetry (DSC), and polymer size with gel permeation chromatography (GPC). FTIR spectra were recorded using a Bruker Alpha spectrometer (Billerica, Massachusetts, USA) with an attenuated total reflectance solid sample holder. DSC was performed using a TA DSC Q2000 from TA Instruments (New Castle, Delaware, USA). Roughly 3 mg of nanocomposite sample was placed in an aluminum Tzero pan (TA Instruments) and sealed with a hermetic lid (TA Instruments). Samples were exposed to the following heating cycle. The temperature was increased from 20 to 170 °C at a rate of 10 °C min^–1^, held at 170 °C for 2 min, decreased from 170 to 20 °C at 10 °C min^–1^ and held at 20 °C for 2 min. These cycles were repeated for a total of three full heating and cooling cycles.

Polymer sizes were measured using a Styragel HR 4 THF GPC column (7.8 mm × 300 mm, Milford, Massachusetts, USA) attached to a Waters 1525 HPLC system (Standards SM-105 from Shodex, Tokyo, Japan). Polymer molecular weights were calibrated with polystyrene standards (Shodex Standards SM-105). Samples were prepared for GPC by dissolving the polymer in 0.05 M LiBr in DMF and filtering through a 0.45 μm-pore syringe filter.

### 2.5. Fluorescence data

All fluorescence measurements were recorded using a Perkin Elmer LS-55 luminescence spectrometer from Perkin Elmer (Watham, Massachusetts, USA). The ability of TiO_2_ to quench the natural fluorescence of the styrene within the printed polymer was tested. Cylinders (2.5 cm diameter, 50 cm height) were printed with no raft. The side of the cylinder that was in contact with the print bed during fabrication was the smoother of the two sides. These procedures do not result in a cylinder that is flat enough for fluorescence measurements. A single drop of acetone was placed on the top of the cylinder and the window from the sample holder was set on top of the drop for 30 min before fluorescence measurements to flatten the cylinder. Performing this step increased the reproducibility of the data. Multiple cylinders were printed for each per cent TiO_2_ in ABS, and each cylinder was measured six times. The cylinder was rotated within the sample holder in between each measurement. Emission spectra were collected with an excitation wavelength of 280 nm with a slit width of 10 nm and the emission slit width was set to 4.5 nm. Emission was scanned from 300 nm to 550 nm. The scan speed was 200 nm min^–1^. The emission spectra were corrected such that the emission goes to zero at 550 nm. The total integrated emission intensity was calculated for each measurement.

To test the photocatalytic properties of TiO_2_ in the printed nanocomposites, we placed one half of a printed dogbone in a scintillation vial and immersed it in 10 ml of a 1 μM aqueous rhodamine 6G solution. The fluorescence of the solution was recorded before and after the sample was exposed to direct sunlight for 4 h. To measure fluorescence, the solution was placed in a quartz cuvette. Emission spectra were collected with a 480 nm excitation with a 10 nm slit width and an emission slit width of 10 nm. The emission was scanned from 500 nm to 650 nm. The total integrated intensity was calculated for each spectrum to determine if the TiO_2_ in the printed materials could photocatalytically degrade the rhodamine 6G. As photogenerated radicals might also affect the ABS polymer, we recorded the FTIR spectra of printed dogbones before and after 4 h of exposure to direct sunlight.

## 3. Results and discussion

While developing our system we performed a number of preliminary experiments and observations. First we had to choose the polymer for making nanocomposites. The two polymers that are typically used in consumer 3D printers are ABS and polylactic acid (PLA). We chose to work with ABS as opposed to PLA for this work because we observed that PLA is more prone to decomposition during extrusion (experimental observation). One observation that we made while printing the TiO_2_-ABS composites is that the standard printing speed (30 mm s^–1^) led to poorly printed structures and often led to clogged print heads. We found piano wire to be the best tool for cleaning these clogs. Reducing the printing speed to 10 mm s^–1^ resulted in consistent, quality structures. We also would like to note the importance of allowing the print heads and printing bed to remain at operating temperature for at least a half an hour before starting a print job. This increased the temperature inside of the printing chamber and reduced the chances that the structure would curl up from the print bed during the print job.

### 3.1. TiO_2_ in ABS

**Figure 1.**
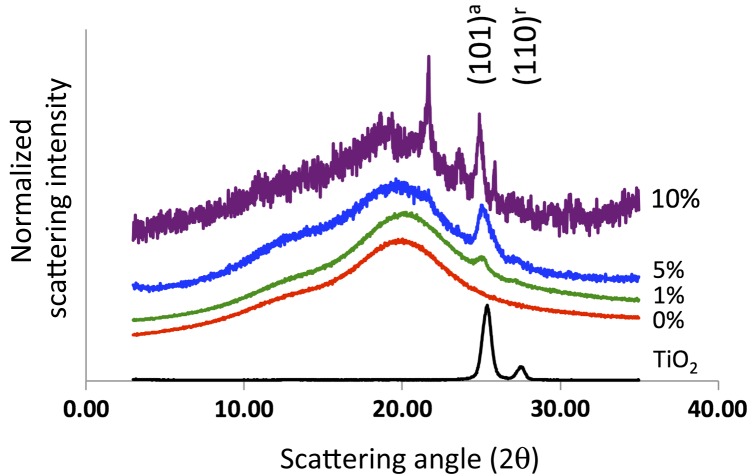
X-ray diffraction spectra of TiO_2_ powder (black, no vertical offset), 0% TiO_2_ in ABS (red, smallest vertical offset), 1% TiO_2_ in ABS (green, small vertical offset), 5% TiO_2_ in ABS (blue, medium vertical offset), and 10% TiO_2_ in ABS (purple, large vertical offset). The scattering signals at 2*θ* = 25.4° and 27.5° correspond to scattering from the anatase and rutile polymorphs, respectively (International Centre for Diffraction Data reference file for anatase TiO_2_: 21–1272 and rutile TiO_2_: 21–1276). The 2*θ* = 25.4° scattering arises from the (101) plane of the anatase polymorph and corresponds to a d-spacing of 0.3504 nm. The 2*θ* = 27.5° scattering arises from the (110) plane of the rutile polymorph and corresponds to a d-spacing of 0.3241 nm.

Figure 1 shows the XRD patterns of TiO_2_, 0% TiO_2_ in ABS (1%), 1% TiO_2_ in ABS (1%), 5% TiO_2_ in ABS (5%), and 10% TiO_2_ in ABS (10%). The 1, 5, and 10% samples show the presence of TiO_2_. The intense peak (at 2*θ* = 25.4°) from the anatase polymorph of TiO_2_ is present in all TiO_2_-ABS samples along with the signal (at 22*θ* = 27.5°) from the rutile polymorph of TiO_2_. The signal of all TiO_2_-associated peaks increases (with respect to the broad ABS signal at 2*θ* ≈ 20°) with increasing percentage of TiO_2_ within the ABS composite. Full spectra (from 2*θ* = 3° to 2*θ* = 90° are shown in the Supporting Information).

### 3.2. Effect of TiO_2_ and processing on ABS structure

ABS is formed when styrene and acrylonitrile are polymerized in the presence of pre-formed polybutadiene creating a network of polybutadiene and poly(styrene-co-acrylonitrile) polymers. The solvent casting, extrusion, and printing steps place a significant amount of strain on the ABS. The presence of TiO_2_ during these processes can exacerbate any damage to the polymer. Because TiO_2_ can photocatalytically produce free radicals,[25] ABS can be degraded and altered throughout the progression from solvent casting to printed structure. To understand how each preparation step affected the polymeric structure, we monitored the FTIR spectra, glass transition temperature, and polymer molecular weight at different points in the processing for all of the TiO_2_-ABS nanocomposites used.

**Figure 2.**
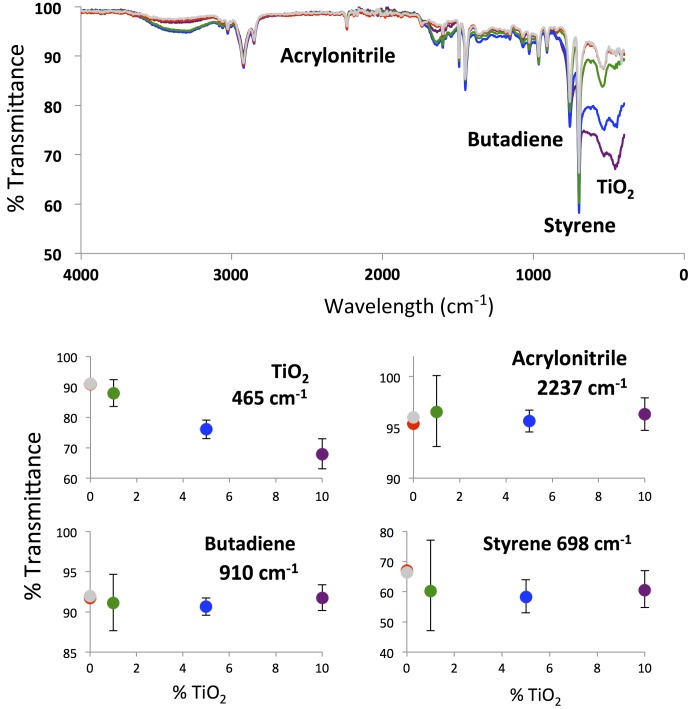
FTIR spectra for printed TiO_2_-ABS composites. The top panel includes the full spectrum for each composite, averaged over 10 printed samples. The bottom panel displays the % transmittance at wavelengths associated with the different components for the TiO_2_-ABS composites. The error bars correspond to the standard deviation from the averaged measurement over all spectra acquired. In these spectra, different colors (for the lines in the upper panel and circles in the bottom panels) correspond to samples printed from different composites. Gray: commercial ABS filament. Red: extruded 0% TiO_2_-ABS composites. Green: extruded 1% TiO_2_-ABS composites. Blue: extruded 5% TiO_2_-ABS composites. Purple: extruded 10% TiO_2_-ABS composites.

FTIR analysis assesses how the individual components (acrylonitrile, butadiene, and styrene) are affected during the processing steps. Figure 2 displays the FTIR spectra for 3D printed samples (dogbones and cylinders). Multiple spectra were recorded for each TiO_2_ percentage. The averaged spectra are shown in the top panel while the bottom panels show the average transmittance with standard deviations for the wavelengths associated with different components of the ABS-TiO_2_ nanocomposite.

The data corresponding to TiO_2_ absorption (absorbance feature at 465 cm^−1^) show increasing amounts of TiO_2_ with increasing TiO_2_ percentage within the nanocomposite. These data are in agreement with the data in Figure 1. The spectra in Figure 2 are taken for printed samples, therefore the data show that TiO_2_ remains within the ABS matrix during the extrusion and printing processes.

The data for acrylonitrile (λ_abs_ = 2237 cm^−1^), butadiene (λ_abs_ = 910 cm^−1^), and styrene (λ_abs_ = 698 cm^−1^) paint a different picture. None of these absorption features differ significantly from the samples printed with commercial ABS filament and lab-extruded (0% TiO_2_) ABS filament. There are no trends observed in these samples with increasing TiO_2_ concentration. This result is to be expected, as the sample matrices are still primarily ABS.

The standard deviations for the acrylonitrile, butadiene, and styrene features associated with the 1% TiO_2_ samples are markedly higher than those of the other samples. The deviation in the 1% samples (±1.7, 9.2, and 16.9% for acrylonitrile, butadiene and styrene, respectively) is clearly larger than the deviation observed for the 5% and 10% TiO_2_ samples. We argue that, as the amount of TiO_2_ is increased in the nanocomposite, the amount of nanoparticle clumping increases. As the clumping increases, the direct net interactions between ABS and TiO_2_ decreases. That is, because some TiO_2_ nanoparticles are sequestered within aggregates, these nanoparticles do not contact the polymer matrix. If TiO_2_ is capable of facilitating polymer degradation, samples with less clumping will increase degradation. We contend that TiO_2_ dispersity in the 1% sample leads to increased polymer–nanoparticle interactions. The increased number of nanoparticle-polymer interactions in the 1% sample lead to changes in absorption features and, potentially, increased polymer degradation during processing. Increased deviations observed for the acrylonitrile, butadiene, and styrene FTIR peaks are due to increased polymer–nanoparticle interactions. This description (increased clumping leading to fewer interactions between the polymer and the nanoparticle) is consistent with other experimental observations, which will be discussed below.

**Figure 3.**
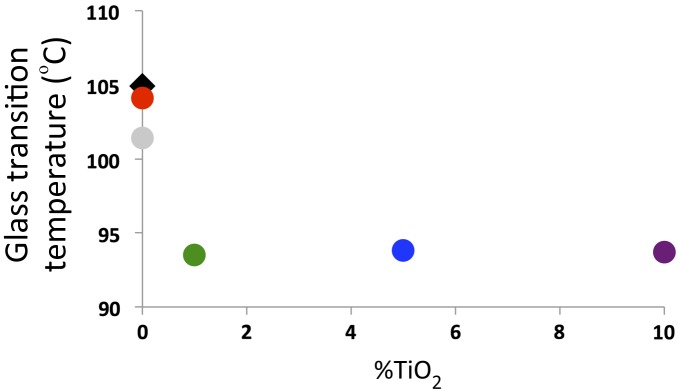
Glass transition temperatures of different samples from printed TiO_2_-ABS nanocomposite filaments. Unprocessed ABS pellet (black diamond). Commercial ABS filament (gray circle). Lab extruded ABS filament (red circle). 1% TiO_2_-ABS filament (green circle). 5% TiO_2_-ABS filament (blue circle). 10% TiO_2_-ABS filament (purple circle).

ABS is naturally amorphous and displays no melting or crystallization features. ABS has a glass transition temperature (T_g_) at roughly 105°C. The T_g_ values of the different nanocomposites are shown in Figure 3. The T_g_ of an unprocessed ABS pellet (unaltered from the supplier) and of the lab-extruded 0% TiO_2_ ABS filament are both near the published value of 105°C. The commercial ABS filament has a lower T_g_ (101°C). The T_g_ values for the 1, 5, and 10% TiO_2_-ABS nanocomposites are all significantly lower than the pure ABS samples. The reduction in T_g_ upon TiO_2_ addition is consistent with other experiments.[32]

The predominant cause for decreased T_g_ in the ABS that contains TiO_2_ is the nanoparticle–polymer interaction. It has been observed that TiO_2_ reduces the glass transition temperatures in polymeric systems where there is poor interfacial adhesion, leading to an increase in free volume and polymer chain mobility near the particle.[32–34] The T_g_ data are consistent with the FTIR spectra. Increasing the dispersion of the nanoparticles within the polymer matrix will increase the total surface area of TiO_2_ available to ABS. If the 5% and 10% samples contained dispersed nanoparticles, the T_g_ values would decrease for these nanocomposites consistent with the amount of TiO_2_ added. We contend that the T_g_ for the 5% and 10% samples do not decrease in comparison to the 1% sample due to nanoparticle aggregation.

The molecular weight of the ABS at different stages of processing was analyzed using GPC. Polystyrene standards were used to correlate retention time to molecular weight. The data for these standards were fit using a quadratic equation (shown in the Supplemental Information) to determine the molecular weight of the processed polymer. Table 1 shows the peak molecular weight (M_n_) and polydispersity index (PDI) data for the TiO_2_-ABS nanocomposites after: solvent casting, extruding through the compounder, extruding into a 1.75 mm filament, and 3D printing.

**Table 1.** Molecular weight and polydispersity index (PDI) determination for ABS after each processing step.

ABS pellet	M_n_ in kDa (PDI) = 210 (1.9)			
	Solvent cast	Extrusion 1	Extrusion 2	Printed
1% TiO_2_	170 (1.9)	170 (1.8)	180 (2.0)	180 (1.9)
5% TiO_2_	180 (1.8)	190 (1.7)	190 (1.8)	190 (1.7)
10% TiO_2_	220 (1.7)	200 (1.6)	180 (1.8)	190 (1.9)

The retention time was determined by selecting the peak maximum in the recorded GPC trace. Molecular weight data were determined with the calibration curve (described above) using the Breeze software that controls the Waters HPLC system. Several samples for each processing step were acquired. The PDI, which is calculated by dividing the molecular weight average (M_w_) by the number average molecular weight (M_n_), is indicative of the molecular weight distribution of the polymers in solution. M_n_ decreases from the value observed for the ABS pellet for each ABS-TiO_2_ sample except for the solvent cast 10% TiO_2_ sample. We argue that the mass difference between the polymer from the ABS pellet and the solvent cast 10% TiO2 sample are statistically insignificant; a variation of 10 kDa is within the expected error of the measurement for the column used here. The change in molecular weight is largest for the 1% sample. This observation is consistent with the result showing that greater dispersion of TiO2 in the 1% sample leads to more ABS decomposition (Figure 2). From the data in this table, it would appear that most of the polymer degradation occurs during the solvent casting process. In this step, ABS is sonicated with TiO_2_ at 37°C in acetone, poured into a Teflon coated pan, and heated at 80°C to evaporate the solvent. The effects of the other processing steps on polymer size seem to be less than the effect of the casting step. To support these data, we have also measured the FTIR spectra of the nanocomposites after each processing step. These spectra are presented in the Supporting Information.

### 3.3. Mechanical properties of the printed TiO_2_-ABS nanocomposites

TiO_2_ has previously been shown to alter the mechanical properties in printed ABS.[22] This type of observation is consistent with other experiments on TiO_2_-polymer nanocomposite materials.[20, 23] As opposed to other nanocomposite structures, 3D printed structures have natural striations resulting from the printing resolution. It is possible that the printing process affects the homogeneity of the nanocomposite. We measured the mechanical properties of the printed materials to test the effect of these qualities and to directly study the effect of TiO_2_ on ABS. ASTM D638 standard dogbones were printed and analyzed using a Series 5 Universal Testing Machine from Mark-10 (Copiague, New York, USA) for stress-strain and three-point bending measurements.

**Figure 4.**
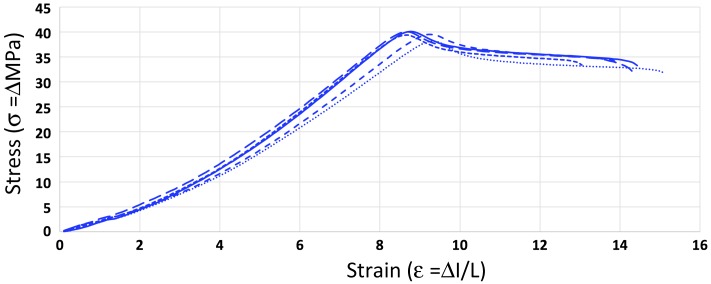
The stress-strain curves for the 5% TiO_2_ ABS nanocomposites. The stress-strain curves for the other composites are shown in Figures S7–S9. The analyzed data for all composites are show in Table 2.

**Figure 5.**
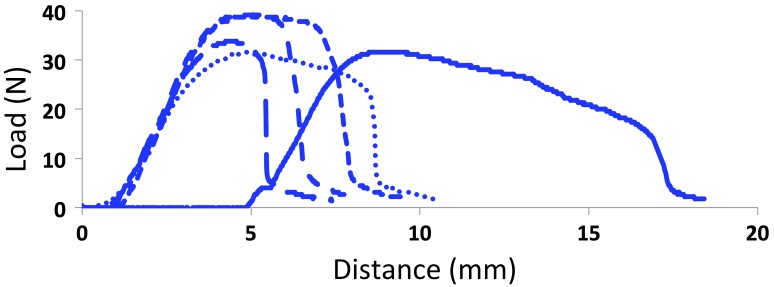
The three-point flexural measurements for the 5% TiO_2_-ABS composite dogbones. The flexural measurements for the other composites are shown in Figures S10–S11. The analyzed data for all TiO_2_-ABS composites are shown in Table 2.

Figure 4 shows the stress-strain curves measured for 5% TiO_2_-ABS dogbones, and Figure 5 shows the flexural testing results of these same materials. Several mechanical properties calculated from these experiments are shown in Table 2. Among these properties are the ultimate tensile strength (UTS), the stress measurement at UTS, Young’s modulus, and the flexural strength. For each of these properties, the average measurements for each nanocomposite percentage are shown along with their deviations.

**Table 2.** Mechanical properties and measurement uncertainties for average stress, average strain, Young’s modulus, and flexural strength of each nanocomposite.

	Mechanical testing			
	Average stress (MPa)	Average strain (%)	Young’s modulus (MPa)	Flexural strength (MPa)
0% TiO_2_	41.4 ± 0.7	9.0 ± 0.2	6.1 ± 0.2	70 ± 2
1% TiO_2_	39.4 ± 0.8	8.9 ± 0.3	6.1 ± 0.5	62 ± 5
5% TiO_2_	43.6 ± 0.2	9.7 ± 0.8	6.2 ± 0.7	72 ± 8
10% TiO_2_	43 ± 1	9.1 ± 0.9	6.5 ± 0.4	72 ± 2

The UTS and flexural strength are both measurements of the extreme forces that a material can handle before it fails. The nanocomposites show similar trends for these values with respect to the amount of TiO_2_ added. The 1% TiO_2_ nanocomposites show a decrease in both UTS and flexural strength as compared to the 0% sample. The 5 and 10% TiO_2_ nanocomposites show an increase in UTS and flexural strength in comparison to the 0 and 1% samples. Young’s modulus values and the stress at material failure do not show any significant variations as a function of TiO_2_ percentages.

We have argued that nanoparticle clumping within the polymer matrix is the cause for variations in glass transition properties as a function of TiO_2_ percentages. We contend that clumping also plays a role in affecting the mechanical properties. The 1% TiO_2_ samples show better dispersion than the 5% and 10% samples, and the 1% samples show lower mechanical strength in both tensile and flexural measurements, therefore the interaction between the polymer and the nanoparticles (21 nm) must have an adverse effect on the strength of the material matrix. As the nanoparticles clump together, there are several possible mechanisms that might lead to an increase in material strength. The first of these possibilities is that the effective size of the TiO_2_ plays a role in determining tensile and flexural strength. As the effective particle gets larger (and the surface area to volume decreases), the total number of particle–polymer interactions decrease. If the interaction itself has an adverse effect on the strength of the matrix, decreasing the number of interactions will increase the strength. This possibility is consistent with the increased polymer flexibility near the nanoparticles that leads to a decrease in T_g_.[33] Another possibility is that individual polymer chains can intercalate between nanoparticles within a single clump. The intercalation might make the polymer matrix stronger. We expect that the strength (justified by either of the two descriptions) will eventually stop increasing as a function of particle clumping, which has also been shown to induce fracture points in nanocomposites with high loading percentages.[20] We should note that in the previous study on 3D printed TiO_2_-ABS structures, the researchers only prepared a 5% TiO_2_ nanocomposite.[22] They observe an increase in tensile strength over the 0% nanocomposite. This observation is consistent with our experiments.

### 3.4. Photocatalytic degradation of rhodamine

TiO_2_ and TiO2 nanocomposites have been shown to generate reactive oxygen-based radicals when exposed to sunlight.[25] These radicals can take part in reactions with organic compounds in close proximity to the nanoparticles. It has been proposed that TiO_2_-containing materials can assist with the degradation of environmental pollutants in both wastewater streams and in the atmosphere. One of the most common tests of the photocatalytic properties of nanocomposites includes immersing the nanocomposite in a solution of fluorescent dye, exposing the mixture to light, and measuring the fluorescence of the solution as a function of time.[24]

**Figure 6.**
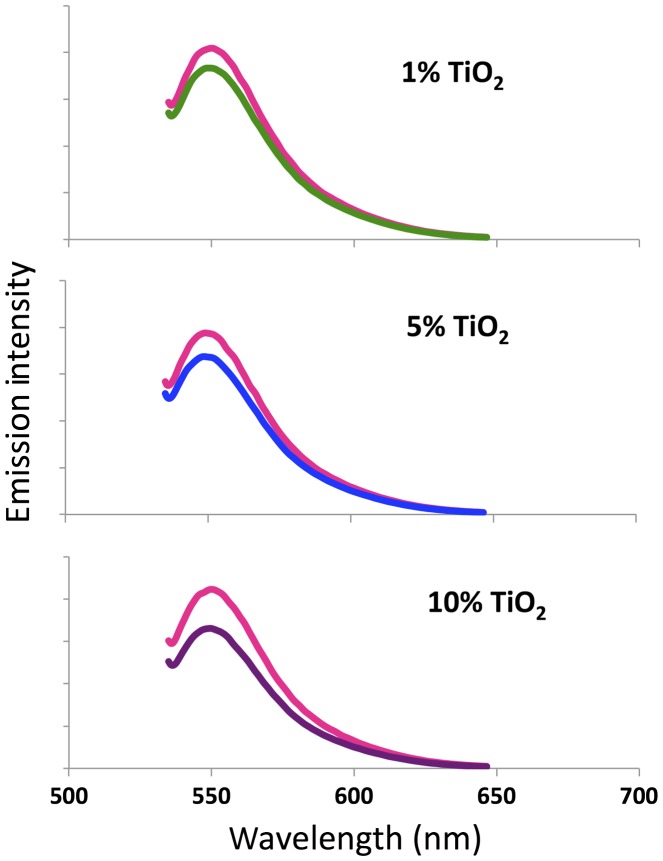
Fluorescence emission spectra of a rhodamine 6G solution before and after exposure to 4 h of sunlight in the presence of different samples of printed TiO_2_-ABS nanocomposites. The pink curve in each plot corresponds to the rhodamine emission at the start of the experiment. The green curve (1% TiO_2_-ABS), blue curve (5% TiO_2_-ABS), and purple curve (10% TiO_2_-ABS) correspond to the spectra recorded after 4 h in direct sunlight.

Figure 6 shows the fluorescence spectra of rhodamine 6G solutions before and after exposure to sunlight in the presence of printed TiO_2_-ABS samples. These spectra show a marked decrease in fluorescence emission over time; and the magnitude of this change increases as a function of TiO_2_ concentration. The integrated intensity for each sample decreases as in the following manner. The 1% sample shows a decrease of integrated fluorescence intensity of 10% over time. The 5% sample shows a decrease of integrated fluorescence intensity of 13% over time. And the 10% sample shows a decrease of integrated fluorescence intensity of 22% over time.

It should be noted that these experiments used 3D printed dogbones. This geometry is hardly ideal for facilitating TiO_2_ photocatalysis, which should include a high surface area to volume structure to increase the interaction of nanoparticles with the dye in solution. In fact we would only expect to see photogenerated radicals from the surface of the printed material. As the matrix that surrounds the TiO_2_ can also be degraded by the photogenerated radicals, we measured the FTIR spectra before and after a 6 h solar exposure. Over this time period, we observe some minimal degradation of the ABS polymer (data shown in the Supporting Information). Because of the promising results described above, we have begun to experiment with different printed geometries to determine an optimal printed shape for applications that involve photocatalytic removal of environmental pollutants.

## 4. Conclusions

We have developed a process for generating TiO_2_-ABS nanocomposite filaments for application in 3D printing. We have reported on the physical, mechanical, and chemical properties of these materials after they have been printed. The analysis of rhodamine 6G degradation characterizes the chemical and catalytic activity of a material that was produced using a commercial thermoplastic 3D printer. We expect to expand upon these studies by incorporating other, chemically active nanoparticles within ABS and bring new chemistry to 3D printed materials.

## Disclosure statement

No potential conflict of interest was reported by the authors.

## Notes on contributors

At the time of publishing, Skorski and Esenther are both undergraduates at American University. Ahmed is on staff at the National Institutes of Standards and Technology whose primary research focus is on materials for photonics sensing. Miller has recently moved from a faculty position at American University to the United State's Food and Drug Administration. Hartings is a faculty member at American University who studies a broad range of polymer-transition metal nanoparticle nanocomposites, which includes areas that vary from biomineralization to synthesizing the types of industrial polymer nanoparticle systems described in this manuscript.
